# Radiation dose and physical image quality in 128‐section dual‐source computed tomographic coronary angiography: a phantom study

**DOI:** 10.1120/jacmp.v13i5.3959

**Published:** 2012-09-06

**Authors:** Kosuke Matsubara, Haruka Koshida, Keita Sakuta, Tadanori Takata, Junsei Horii, Hiroji Iida, Kichiro Koshida, Katsuhiro Ichikawa, Osamu Matsui

**Affiliations:** ^1^ Department of Quantum Medical Technology Faculty of Health Sciences Kanazawa University Kanazawa; ^2^ Department of Radiological Technology Kanazawa University Hospital Kanazawa; ^3^ Department of Radiology Faculty of Medicine Kanazawa University Kanazawa Japan

**Keywords:** computed tomography, coronary angiography, radiation dosage, image quality

## Abstract

One‐hundred‐and‐twenty‐eight–section dual X‐ray source computed tomography (CT) systems have been introduced into clinical practice and have been shown to increase temporal resolution. Higher temporal resolution allows low‐dose spiral mode at a high pitch factor during CT coronary angiography. We evaluated radiation dose and physical image qualities in CT coronary angiography by applying high‐pitch spiral, step‐and‐shoot, and low‐pitch spiral modes to determine the optimal acquisition mode for clinical situations. An anthropomorphic phantom, small dosimeters, a calibration phantom, and a microdisc phantom were used to evaluate the radiation doses absorbed by thoracic organs, noise power spectrums, in‐plane and z‐axis modulation transfer functions, slice sensitivity profiles, and number of artifacts for the three acquisition modes. The high‐pitch spiral mode had the advantage of a small absorbed radiation dose, but provided low image quality. The low‐pitch spiral mode resulted in a high absorbed radiation dose of approximately 200 mGy for the heart. Although the absorbed radiation dose was lower in the step‐and‐shoot mode than in the low‐pitch spiral mode, the noise power spectrum was inferior. The quality of the in‐plane modulation transfer function differed, depending on spatial frequency. Therefore, the step‐and‐shoot mode should be applied initially because of its low absorbed radiation dose and superior image quality.

PACS numbers: 87.57.‐s; 87.57.C‐; 87.57.cf; 87.57.cm; 87.57.cp; 87.57.Q‐; 87.57.qp; 87.57.uq

## I. INTRODUCTION

Modern computed tomography (CT) systems employ multidetector arrays that allow rapid acquisition and wide coverage.^(1)^ The introduction of 16‐section multidetector row CT (MDCT) systems has led to improved spatial and temporal resolution.^2^, ^3^ In addition, CT coronary angiography (CTCA) has evolved as a noninvasive tool with high accuracy for assessing coronary artery disease since the implementation of 64‐section MDCT systems.^4^, ^5^

Generally, CTCA is performed applying two acquisition modes: retrospective electrocardiogram (ECG)‐gated low‐pitch spiral (LPS) mode and prospective ECG‐triggered step‐and‐shoot (SAS) mode. In the LPS mode, CT data are acquired throughout the cardiac cycle, and the data required for the reconstruction phase are chosen retrospectively.^(6)^ In the SAS mode, CT data are only acquired over a fraction of the R–R interval.^(6)^ The SAS mode results in low radiation exposure during CTCA and provides high diagnostic accuracy in the assessment of coronary artery disease; however, it can be applied only in patients with stable sinus rhythms and low heart rates.^(7)^ In contrast, the LPS mode can be applied to patients with high heart rates; however, the radiation doses received by patients are considerably higher than those received in SAS mode because of a low pitch factor.^(8)^

In recent years, new CT systems equipped with dual X‐ray sources (DS) in one gantry have been introduced into clinical practice and found to increase temporal resolution compared with that of a single‐source system.^9^, ^13^ The higher temporal resolution of a 128‐section DSCT allows the use of a prospective ECG‐triggered spiral mode at a high pitch factor.^12^, ^13^ Achenbach et al.^(13)^ also reported that prospective ECG‐triggered high‐pitch spiral (HPS) CTCA provided excellent image quality at a consistent dose below 1.0 mSv in nonobese patients with low and stable heart rates. Although the effective dose is regarded as the best available dose descriptor for quantifying stochastic risks in diagnostic radiology, one previous study demonstrated that effective doses were comparatively lower than doses absorbed by certain pericardial tissues on CTCA, and that it was preferable to indicate measurable organ‐absorbed doses at the same time.^(14)^ In addition, evaluation of the physical image qualities, such as the noise power spectrum (NPS),^15^, ^16^ modulation transfer function (MTF),^17^, ^18^ slice sensitivity profile (SSP),^19^, ^20^ and the number of artifacts, is also important to confirm the effectiveness of HPS, SAS, and LPS modes.

The aim of this study was to measure organ‐absorbed doses and evaluate the physical image quality of 128‐section DS CTCA in HPS, SAS, and LPS modes to confirm which mode should be applied regularly in clinical situations.

## II. MATERIALS AND METHODS

### A. CT system and phantom

A 128‐section DSCT SOMATOM Definition Flash (Siemens Healthcare, Erlangen, Germany) was used in this study.

**Figure 1:**
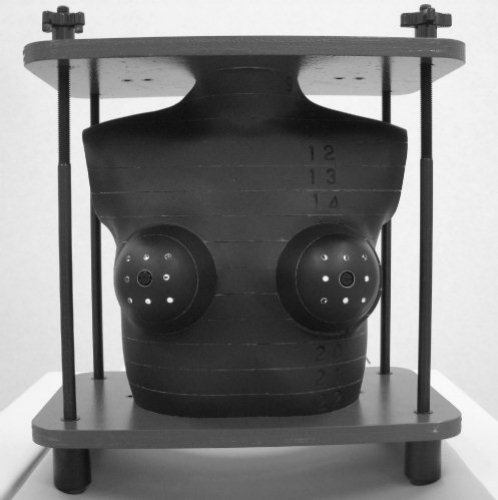
The anthropomorphic female thoracic phantom used in this study. The entire phantom was cut into thin transverse sections with grids of holes for placing small dosimeters.

For dose evaluation, we used the RAN‐110 (Phantom Laboratory, Salem, NY) anthropomorphic female thoracic phantom onto which two breast sections were mounted (Fig. 1). The assembly included an embedded natural skeleton, epoxy resin‐based lung substitute, and an isocyanate rubber‐based muscle substitute.^(21)^ We used the Catphan 600 calibration phantom (Phantom Laboratory) and the MHT microdisc phantom (Kyoto Kagaku, Kyoto, Japan) to evaluate the physical image qualities of the MDCT scanners.

### B. Dosimeters and dose calibration

Radiophotoluminescent glass dosimeters (RPLDs) (GD‐302M; Chiyoda Technol, Tokyo, Japan) were used to estimate the radiation dose absorbed by the organs. A $3\;{\text{cm}}^{3}$ ion chamber attached to a 120 kVp (effective energy 50 keV) diagnostic X‐ray beam was used to perform dose calibration against an ionizing dosimeter (Ramtec 1500B; Toyo Medic, Tokyo, Japan). The chamber and RPLDs were placed side‐by‐side at the same distance from the X‐ray focus in an irradiated field. The ionizing dosimeter had been calibrated at a laboratory accredited by the Japan Quality Assurance Organization. The RPLDs were annealed at 400°C for 30 min before each exposure. After each exposure, the RPLDs were further heated to 70°C for 30 min and read by an FGD‐1000 reader (Chiyoda Technol) in accordance with the manufacturer's recommended protocol.

### C. Measurement of the organ‐absorbed dose

After obtaining localization radiographs, we placed 48 RPLDs at locations that corresponded to those of the breast (six RPLDs); heart (eight RPLDs); lung (eight RPLDs); red bone marrow within the ribs, sternum, and thoracic vertebrae (six, two, and four RPLDs, respectively); thymus (two RPLDs); and skin (12 RPLDs). Two RPLDs were used to measure the background radiation.

Thereafter, prospective ECG‐triggered HPS mode (Flash Spiral Cardio; Siemens Healthcare), prospective ECG‐triggered SAS mode (Flash Sequence Cardio; Siemens Healthcare), and retrospective ECG‐gated LPS mode were applied at a fixed heart rate of 60 beats per minute (bpm). Exposure parameters (Table 1) were selected according to those used in our institution. An ECG monitor demo mode function was used to simulate an arbitrary heart rate of 60 bpm. Each measurement was performed four times to reduce random errors.

**Table 1:** Acquisition parameters for evaluating absorbed doses and physical image quality.

	*Acquisition Mode*		
*Parameter*	*High‐pitch Spiral*	*Step‐and‐Shoot*	*Low‐pitch Spiral*
Collimation (mm)	128×0.6	128×0.6	128×0.6
kV	120	120	120
Pitch	3.4:1	N/A	0.17:1
mAs	340	340	340
Rotation time (s)	0.28	0.28	0.28
Scan range (mm)	153	172	153
Padding window (%)^a^	N/A	35–85	N/A
Scan duration (s)	0.4	7.0	8.0

N/A=not applicable.

^a^ Acquisition with longer periods of active tube current can be performed by means of variable extension of the padding window, which allows assessment of the heart in several phases of the cardiac cycle.

Subsequently, the absorbed dose for each organ was calculated by multiplying the calibrated mean dose values obtained from the reader by the mass energy coefficient ratio of each organ to air.^(22)^ For the thymus, we used the coefficient of lymph nodes.

### D. Measurement of the NPS

The reconstructed images of the image uniformity module, which were scanned at the isocenter by measuring NPS, were used to evaluate the noise properties in each acquisition mode. A displayed field of view (DFOV) of 220 mm, a slice thickness of 0.75 mm, and a reconstruction kernel of B35f (HeartView medium) were used to reconstruct all images. Exposure parameters were the same as those indicated in Table 1.

To calculate NPS, the central 256×150 pixels in the reconstructed images were used. The Fourier transforms of one‐dimensional noise profiles obtained by the numerical slit scanning technique were used to calculate one‐dimensional NPS.^(23)^ In this technique, five slits with a height of 30 pixels (1×30 pixels) were used. To improve the accuracy of NPS data, three different CT images were used to calculate an average NPS.

### E. Measurement of the in‐plane MTF

The reconstructed images of the point‐source bead module, which were scanned at the isocenter by measuring the in‐plane MTF, were used to evaluate the in‐plane spatial resolution of each acquisition mode. The bead was made of tungsten carbide, had a diameter of 0.28 mm, and was positioned 20 mm above the center of the phantom along the y‐axis. To avoid aliasing errors, the images were reconstructed so that the bead was located at the center of the images with a DFOV of 50 mm (sampling pitch, 0.098 mm), a slice thickness of 3.0 mm, and a reconstruction kernel of B35f (HeartView medium). The exposure parameters were the same as those indicated in Table 1.

To calculate the in‐plane MTF, a slit with a height of 40 pixels (1×40 pixels) was used to obtain one‐dimensional profiles that were equivalent to line spread functions (LSFs). The bases of the LSFs were normalized to zero to eliminate noise dependency. Subsequently, the in‐plane MTF was calculated by one‐dimensional Fourier transformation of the LSFs. To improve the accuracy of the MTF data, three different CT images were used to calculate the average MTF.

### F. Measurement of the SSP and z‐axis MTF

Reconstructed images of the MHT microdisc phantom, which were scanned at the isocenter by measuring the SSP and z‐axis MTF, were used to evaluate the slice width and z‐axis spatial resolution of each acquisition mode. The metallic disc had a diameter of 1 mm and a thickness of 0.05 mm. A slice thickness of 0.75 mm, a reconstruction interval of 0.1 mm (equal to the sampling pitch) to avoid aliasing errors, a DFOV of 50 mm, and a reconstruction kernel of B35f (HeartView medium) were used to reconstruct the images. The reconstructed location was adjusted so that the disc was located at the center of the images. The exposure parameters were the same as those indicated in Table 1.

To calculate the SSP, an oval region of interest, which had a diameter of 12 pixels (approximately 1.2 mm), was placed over the center of the images, and the z‐axis profile was obtained. The bases of the z‐axis profile were normalized to zero to eliminate the noise dependency. Subsequently, an interpolation method was used to calculate the full width at half maximum (FWHM) and full width at tenth maximum (FWTM) of each mode.

The obtained z‐axis profiles were equivalent to the LSF values. Therefore, the z‐axis MTF was calculated by one‐dimensional Fourier transformation of the LSF values. To improve the accuracy of MTF data, three different CT images were used to calculate the average MTF.

### G. Evaluation of artifacts

The reconstructed images of the high‐resolution module with a 21 line pairs per cm gauge, which were scanned at the isocenter, were used to subjectively evaluate the number of artifacts in the images for each acquisition mode. The gauge was cut from 2.0‐mm thick aluminum sheets and cast in an epoxy resin.

Beam‐hardening artifacts appeared as dark bands or streaks. There is a possibility that these artifacts were caused by the gauge, which was cut from aluminum sheets, and we believed that they helped to increase the number of artifacts.

A DFOV of 220 mm, a slice thickness of 0.75 mm and a reconstruction kernel of B35f (HeartView medium) were used to reconstruct all images. The exposure parameters were the same as those indicated in Table 1.

### H. Software

ImageJ (National Institutes of Health, Bethesda, MD) and Excel 2010 (Microsoft, Redmond, WA) were used to evaluate the physical image quality.

## III. RESULTS

**Figure 2:**
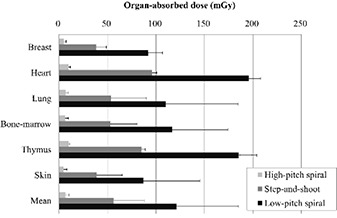
Comparison of organ‐absorbed doses between the three acquisition modes at an assumed heart rate of 60 beats per minute. Bars represent the average dose of the multiple radiophotoluminescent glass dosimeters placed at locations corresponding to those of each organ. Error bars are 2 standard deviations.

The differences in the absorbed doses for each organ received in the HPS, SAS, and LPS modes at a fixed heart rate of 60 bpm are shown in Fig. 2. The highest absorbed doses were observed for the heart: 10.0, 96.1, and 195.7 mGy for the HPS, SAS, and LPS modes, respectively. The doses absorbed by the breast were approximately half of those absorbed by the heart: 5.0, 38.4, and 92.0 mGy for the HPS, SAS, and LPS modes, respectively. The standard deviations of the absorbed doses for the lung, red bone marrow, and skin were relatively high because these organs have large volumes. The entirety of these organs was not included within the acquisition range.

**Figure 3:**
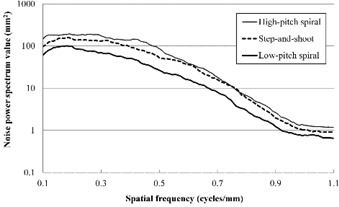
Noise power spectrum results for the three acquisition modes. Each graph was drawn by simply calculating the average of 12 points.

The results of the NPS measurements for the three acquisition modes are shown in Fig. 3. The NPS values for the LPS mode were superior to those for the HPS and SAS modes. The NPS values for the SAS mode were slightly superior to those for the HPS mode.

**Figure 4:**
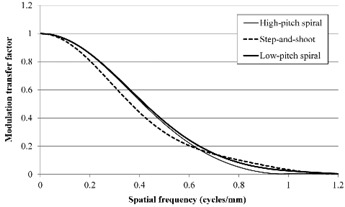
In‐plane modulation transfer function results for the three acquisition modes.

The results of in‐plane MTF measurement for the three acquisition modes are shown in Fig. 4. The MTF for the LPS mode was slightly superior to that for the HPS mode. The MTF values less than 0.6 cycles/mm for the SAS mode were inferior to those for the HPS and LPS modes, but the MTF values greater than 0.7 cycles/mm for the SAS mode were superior to those for the HPS and LPS modes. The values for 50% MTF were 0.41, 0.36, and 0.42, and the values for 10% MTF were 0.73, 0.80, and 0.77 for the HPS, SAS, and LPS modes, respectively.

**Figure 5:**
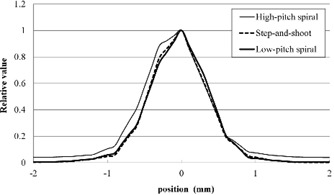
Slice sensitivity profile results for the three acquisition modes.

The results of SSP measurement for the three acquisition modes are shown in Fig. 5. The FWHM values were 0.92, 0.85, and 0.87 mm, and the FWTM values were 1.88, 1.64, and 1.66 mm for the HPS, SAS, and LPS modes, respectively. The error margins around the nominal value of the slice thickness (0.75 mm) for the FWHM were 23.2, 13.5, and 16.4% for the HPS, SAS, and LPS modes, respectively.

**Figure 6:**
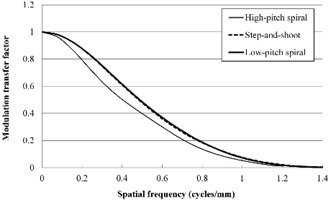
Z‐axis modulation transfer function results for the three acquisition modes.

The results of the z‐axis MTF measurement for the three acquisition modes are shown in Fig. 6. The z‐axis MTFs for the SAS and LPS modes were superior to those for the HPS mode. The values for 50% MTF were 0.40, 0.48, and 0.49, and the values for 10% MTF were 0.87, 0.95, and 0.94 for the HPS, SAS, and LPS modes, respectively.

**Figure 7:**
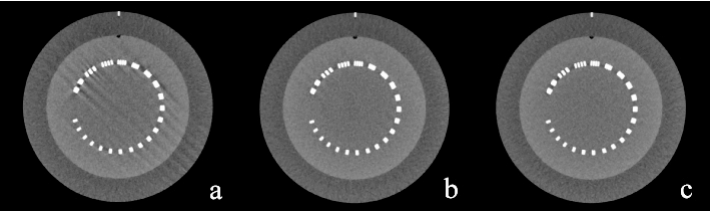
Reconstructed images used for evaluation of the number of artifacts: (a) high‐pitch spiral mode, (b) step‐and‐shoot mode, and (c) low‐pitch spiral mode. The viewing window settings of these images with a window width of 500 Hounsfield units (HU) and a window level of 50 HU.

The reconstructed images of the high‐resolution module with a 21 line pairs per cm gauge, which were scanned at the isocenter, are shown in Fig. 7. The number of artifacts in the reconstructed image for the HPS mode was larger than those for the other two acquisition modes.

## IV. DISCUSSION

We evaluated three acquisition modes of 128‐section DS CTCA. The HPS mode had the advantage of small absorbed dose but gave inferior image quality. The absorbed radiation dose was considerably lower for the SAS mode than for the LPS mode. A comparison of the SAS and LPS modes showed that there were advantages and disadvantages in both acquisition modes regarding image quality.

Some studies have evaluated the radiation dose and image quality of HPS mode.^24^, ^25^ Flohr et al.^(24)^ reported that HPS mode (pitch=3.2) produced spatial and low‐contrast resolution, CT number accuracy and linearity, SSPs, image uniformity, and noise equivalent to those of LPS mode (pitch=1.0). Goetti et al.^(25)^ reported that organ‐absorbed and effective doses were significantly lower in HPS mode compared with those in SAS and LPS modes. Our study provides new results based on a different set of phantoms and dosimeters.

In the HPS mode, the heart absorbed the highest doses among all thoracic organs, but the absorbed dose was only 10.0 mGy, which was less than that absorbed during a routine chest C T. ^26^, ^27^ However, the absorbed organ doses were considerably higher for the other two acquisition modes, especially for the heart, which received approximately 200 mGy in the LPS mode. However, we did not use ECG modulation for LPS mode in this study. According to our previous study, LPS mode with ECG modulation reduced doses absorbed by thoracic organs by 6.4% (3.4%–9.2%) compared with those for LPS mode without ECG modulation.^(28)^ Incidentally, for the HPS mode in our study, the effective dose estimated by the dose–length product and a conversion factor (0.014 for chest CT in adults^(1)^) was 1.6 mSv, which was larger than that observed by Achenbach et al.^(13)^ because they applied a lower tube voltage of 100 kV than that used in our study.

However, there were some limitations regarding image quality for the HPS mode. First, the amount of noise was larger than that observed in the other two acquisition modes. Hence, there is a possibility that it cannot be applied to patients with large physiques. Second, spatial resolution for the in‐plane direction approximately greater than 0.6 cycles/mm and for the z‐axis direction was inferior to those for the other two acquisition modes. Because high in‐plane and z‐axis spatial resolution is required for imaging of the heart and coronary arteries,^(29)^ the inferior spatial resolution compared with those for the other two acquisition modes is a serious limitation. Finally, the number of artifacts in the reconstructed image was obviously larger than that for the other two acquisition modes. These artifacts made it difficult to use CTCA to accurately assess the degree of luminal narrowing when heavily calcified plaques were present.^(30)^ Although Srichai et al.^(31)^ reported that HPS mode had image quality no worse than that of LPS mode and there was no significant difference in the inter‐reader variability in diagnosis between the HPS and LPS modes, they excluded patients who had a history of coronary artery bypass surgery or metallic prosthetic valve replacement surgery from the study population. If these patients were to be included in a study population, there could be some deterioration in the quality of clinical images, which has some influence on clinical interpretation because these patients have heavily calcified plaques or metallic valves that cause beam‐hardening artifacts and lead to misdiagnosis.

The NPS of the SAS mode was inferior to that of the LPS mode, but the quality of the in‐plane MTF differed depending on spatial frequency. The number of artifacts hardly differed between the two acquisition modes. However, the absorbed radiation doses for thoracic organs when the SAS mode was applied were considerably lower than those when the LPS mode was applied, even for maximum X‐ray expose over a wide R–R range that included the systole and diastole phases in the SAS mode.

Therefore, each acquisition mode should be selected appropriately. When the HPS mode is applied, it is necessary to carefully consider the risk for deterioration of image quality. The SAS mode should be selected first, after the decrease in exposed dose and increase in image quality have been considered and judged to be suitable; we think that it should be applied to patients with stable sinus rhythm. The LPS mode should be selected only if the other two acquisition modes are difficult to apply. When LPS mode is applied, methods for reducing the radiation dose received by the patient are necessary; these include adjusting the tube current according to a patient's anthropometric data and using the tube current modulation technique.

There were several limitations of our study. First, we used one specific anthropomorphic phantom. The absorbed dose for each organ differed according to the size, shape, and composition of each phantom. Therefore, it is crucial to use other anthropomorphic phantoms to perform similar examinations. Second, we did not estimate the absorbed radiation dose for each organ when low‐kV CTCA was performed. Recently, several studies regarding low‐kV CTCA have been published that reported higher contrast‐to‐noise ratios and reduced radiation doses in diagnostic imaging and the feasibility of using the technique in patients of normal weight.^4^, ^32^, ^33^ Third, although we evenly distributed a limited number of RPLDs at locations that corresponded to those of each organ, the obtained and actual radiation doses absorbed by each organ could differ in actual clinical situations. Fourth, we only simulated a heart rate of 60 bpm, and hence, no data are available for other heart rates. Finally, we did not simulate the motion effect, which is very common in CTCA, because we used a calibration phantom and a microdisc phantom for evaluating the physical image qualities.

## V. CONCLUSIONS

Acquisition modes should be selected appropriately, and the SAS mode should be applied first to patients with stable sinus rhythm because of its low absorbed dose and superior image quality.

## ACKNOWLEDGMENTS

We would like to thank Hisashi Nishibayashi, Katharina Otani, Tomoko Fujihara, and Takeyoshi Nozawa of Siemens Japan K.K. for their kind support. This work was supported by a Grant‐in‐Aid for Young Scientists (B) from the Ministry of Education, Culture, Sports, Science and Technology of Japan (MEXT) (21791175).
